# Concussion symptom clusters and changes in symptom reporting and severity following multiple concussions in male professional rugby league players

**DOI:** 10.1016/j.jsampl.2026.100155

**Published:** 2026-09-24

**Authors:** Martin Schutte, Cameron Owen, Lauren Fortington, Gordon W. Fuller, Ben Jones, Andrew J. Gardner

**Affiliations:** a Specialist Sports Medicine Centre, Canberra, Australia; b Carnegie Applied Rugby Research (CARR) Centre, Carnegie School of Sport, Leeds Beckett University, Leeds, United Kingdom; c England Performance Unit, Rugby Football League, Manchester, United Kingdom; d Sydney School of Health Sciences, The University of Sydney, Camperdown, New South Wales, Australia; e University of Sheffield, Sheffield, United Kingdom; f Division of Physiological Sciences, Department of Human Biology, Faculty of Health Sciences, The University of Cape Town and the Sports Science Institute of South Africa, Cape Town, South Africa; g School of Behavioural and Health Sciences, Australian Catholic University, Brisbane, Australia; h PREM Rugby Limited, London, United Kingdom

**Keywords:** Rugby league, Concussion symptoms, Injury surveillance, Return to play

## Abstract

**Background:**

Symptom reporting remains an important part of the diagnosis and management of concussion. Understanding the patterns of symptoms and symptom clusters in athletes with multiple concussions may be clinically valuable. The purpose of this study was to characterise acute symptom reporting in players with multiple concussions.

**Methods:**

National Rugby League (NRL) players who have sustained multiple concussions between the 2017 and 2022 seasons were included in the study. Data included the symptoms reported on the Sport Concussion Assessment Tool – 5th Edition (SCAT5) during the Head Injury Assessment by players who were subsequently diagnosed with concussion. K-means clustering with k = 6 centres was performed on symptom severity data. Each concussion episode was assigned to one symptom cluster based on the athlete's symptom presentation. Transitions between symptom clusters from initial to subsequent concussions were visualised using a Sankey plot.

**Results:**

There were 120 players who sustained more than one concussion, including 75 players with two, 23 with three, 15 with four, and 7 players with five or more medically diagnosed concussions. The average duration between concussions was 441 days (SD: 332; Md: 370; range: 11–1435 days). There were 6 different symptom clusters identified. Players were commonly found to transition between clusters for subsequent concussions, most commonly the ‘Asymptomatic’ cluster. There was no relationship found for days between concussions and symptom count or severity. Symptom severity, but not symptom count, was associated with days until medical clearance.

**Conclusion:**

Acute symptom reporting varies with subsequent concussions, making it difficult to predict post-concussion symptoms in individual players. Acute symptom severity was associated with time to medical clearance, whereas the number of concussions in the preceding two years was not.

## 1 Introduction

Concussion is a common injury in rugby league across all levels of the game [[1], [2], [3]]. In the Australian professional men's competition, the NRL, the reported incidence is approximately 14 concussions per 1000 player match hours [4], similar to that observed in the English professional men's club competition [2]. Given the high concussion incidence, and the fact that symptom severity at presentation is the most consistent predictor of slower recovery [5], understanding how symptoms present and evolve across multiple episodes in this population is therefore clinically important.

Symptom reporting is the cornerstone of concussion identification, evaluation, and management in the absence of robust objective diagnostic tools. [6] The Sport Concussion Assessment Tool (SCAT), the most widely used standardised concussion assessment tool [6], captures symptoms through a 22-item post-concussion symptom scale (PCSS) in which athletes self-report the presence and severity of symptoms [7]. An exploratory factor analysis of the PCSS identified four distinct symptom clusters, Cognitive Problems and Slowing (7 items), Sleep Problems (2 items), Emotionality (4 items), and Somatic Symptoms (8 items), each considered to reflect a different subtype of concussion [8].

What remains unclear is how symptom reporting changes across multiple concussion episodes. Current evidence is inconsistent. Some studies report greater emotional and somatic symptom severity with repeat concussion but no cumulative effect on cognitive symptoms or recovery duration [[9], [10], [11]],while others find no significant differences in symptom burden or recovery outcomes between first and subsequent concussions [12]. No research has examined whether athletes shift between symptom clusters across episodes, as opposed to worsening within them, and none of this work has been conducted with professional rugby league players.

Understanding how symptom cluster membership changes across concussion episodes has direct clinical relevance: if clusters shift with repeat injury, assessment and management approaches may need to better account for players' concussion history, particularly in informing return-to-play decisions where symptom burden plays a central role [5]. This study therefore had two aims: first, to identify clusters in acute symptom reporting across all concussions recorded during the study period; and second, among players who sustained more than one concussion during that period, to describe whether symptom cluster membership, symptom severity, and return-to-play duration differed between their first and subsequent concussions.

## 2 Methods

### 2.1 Setting and data source

The SCRIMMAGE (Studying Contact Replays: Investigating Mechanisms, Management, and Game Exposures) research program, is an independent, retrospective video review and clinical analysis database; full details have been published [13]. The research program was endorsed by the Rugby League Research Committee. This study was approved by the Institutional Human Ethics Committee (Ref No. H-2012-0344). In accordance with the NRL and Rugby League Players Association (RLPA) Collective Bargaining Agreement, all players consented a priori to the use of deidentified injury data for the use in NRL endorsed research. As such, no individualised written informed consent for the use of a participant's data in this study was sought. Participants were able to withdraw their data from the study if requested. This study was conducted independently of the NRL (i.e., no funding was sought or provided).

Concussion management in the NRL has changed over time following the initial introduction of a Sideline Injury Surveillance (SIS) system during the 2014 finals series. The SIS was expanded in 2015 with additional camera angles, and then added high-definition video at all venues through 2016–2017. Hawkeye was installed at eight venues by late 2017, and in all venues in 2018. Dedicated concussion spotters were trialled in 2018 and, from 2019, potential head injuries were reviewed through a central Injury Surveillance Bunker (ISB) to support Club Medical Officers [13]. The system was reconfigured and centralised to the ISB in 2021, and in 2022 an ISB Medical Officer was introduced with authority to halt play for removal of a player for a head injury assessment.

Briefly, SCRIMMAGE focuses on the NRL, the premier professional club rugby league competition in Australia and New Zealand and includes all players from the 2017–2022 seasons. The NRL concussion management protocol includes sideline video review and an off-site centralised injury surveillance centre where professionals observe all games live, identifying head impacts and potential head injuries. When indicated, players are removed from play for a medical evaluation, known as the Head Injury Assessment (HIA), performed by club medical staff. The HIA includes the SCAT (SCAT5 for the time period included), which incorporates a 22-item post-concussion symptom scale (PCSS). The results of the in-game HIA are retrospectively added to the SCRIMMAGE database at the conclusion of each season.

### 2.2 Clinical data and measures

Data were drawn from the SCRIMMAGE database for two purposes aligned with the study aims (see Table 1). For the first aim, symptom data from the in-game HIA were used for all concussions within the study period to establish symptom clusters, symptom severity, and return-to-play duration. For the second aim, players who sustained a subsequent concussion within two seasons of their first recorded concussion were included to describe differences in symptom cluster membership, symptom severity, and return-to-play duration across concussion episodes.

The SCAT5 [7] includes a 22-item symptom scale and was developed for the health practitioners to use in the evaluations of individuals aged 13 years and over. Symptoms self-reported as being present are also rated for their level of severity on a dimensional scale from 0 (none) to 6 (severe). All items are summed to create an overall symptom severity score (range: 0–132). Information related to the date of the concussion event, and data on the time to medical clearance were recorded for analysis. The date of medical clearance was also recorded for analysis (measured in days post-concussion).

### 2.3 Statistical analyses

All analyses were performed using R (version 4.3.2; R Core Team 2023). Symptom profiles were characterised using k-means clustering on symptom severity data (22 symptoms from the SCAT assessment). The optimal number of clusters was determined using the silhouette method (cut-off <0.4), using the *factoextra* package. K-means clustering with k = 6 centres was performed. Each concussion episode was assigned to one symptom cluster based on individuals' symptom presentation. Transitions between symptom clusters from initial to subsequent concussions were visualised using a Sankey plot. Descriptive labels of the dominant symptoms and severity scores within each cluster are provided to assist readability and interpretation. For players with multiple concussions, each transition is represented within the plot. Discrete-time Markov chain analysis was then used to model transitions between symptom clusters across consecutive concussions, using the *markovchain* package. Analysis was restricted to athletes with multiple concussions. Transition probabilities were estimated using maximum likelihood, where $\hat{p}$ ᵢⱼ = nᵢⱼ/Σ_k_nᵢ_k_, with nᵢⱼ representing observed transitions from cluster i to cluster j. We assumed first-order Markov property (future cluster depends only on current cluster) and time–homogeneous transitions (constant probabilities across concussion episodes). Generalised linear mixed models (GLMMs) were used to evaluate relationships between variables via the *glmmTMB* package.

Separate models evaluated: (1) the relationship between inter-concussion interval (days between consecutive concussions) on symptom burden (i.e., symptom number and severity); (2) the relationship between symptom burden on days to medical clearance; and (3) the relationship between concussion experienced within the last 730 days (2 years) on days to medical clearance. Concussion number was categorised as 0, 1, 2, 3+ to account for small sample sizes in higher concussion counts and to maintain participant anonymity. Models included random intercepts for athletes to account for repeated measures within individuals. For model 1, random intercepts were removed due to a lack of variance explained by the athlete random effect. Models were first fitted using a Poisson distribution; overdispersion resulted in negative binomial distributions being applied for models 1 and 2. Model assumptions were verified using diagnostic plots from the *performance* package.

For models 1 and 2, model coefficients were exponentiated to obtain rate ratios, representing the multiplicative change in the dependent variable for each one-unit increase in the independent variable. Ninety-five percent confidence intervals (95% CIs) were calculated using the Wald method (coefficient ± 1.96 × standard error, exponentiated). Statistical significance was assessed using Wald z-tests, with p-values <0.05 considered statistically significant.

For model 3, the period in which the concussion occurred (before or after 2020) was included as a covariate to account for the change in the minimum mandatory stand-down period. Estimated marginal means for the day to medical clearance and pairwise comparisons were calculated using the *emmeans* package. For each comparison, rate ratios and 95% CIs were computed using the Wald method, and statistical significance was assessed using z-tests. To control for multiple comparisons, the Tukey adjustment was applied. Data are presented as a violin plot with median and interquartile range due to the categorical nature of the number of concussions in the previous 2 years due to the “3+” category.

Finally, we examined whether changes in medical practice, reflective of changing cultures and knowledge over the time, may have influenced our findings [14,15]. A negative binomial regression model was used to examine whether days to medical clearance following concussion diagnosis varied as a function of assessment date. Negative binomial regression was selected over Poisson regression to account for overdispersion in the count outcome. Assessment date was converted to a continuous numeric variable and centred, producing a scaled predictor with a value of zero at the mean assessment date. A linear model including a random intercept for player to account for repeated concussion assessments within individuals was utilised over a fixed effect only and quadratic model. Model selection was conducted using the Akaike Information Criterion (AIC). All models were estimated using the glmmTMB package in R. Model coefficients are reported as incidence rate ratios (IRRs) with 95% confidence intervals, derived by exponentiating the log-linear model estimates and their associated Wald-based standard errors. Given the small magnitude of the daily IRR, the effect of assessment date is additionally expressed per calendar year (365 days) to aid clinical interpretation. Statistical significance was evaluated using Wald z-tests, with a threshold of p < 00.05.

## 3 Results

Clinically diagnosed concussions (n = 430) with accompanying in-game SCAT symptom reporting, sampled across six NRL seasons (2017–2022), were evaluated in this study. There were 111 players diagnosed with one concussion and 120 players diagnosed with more than one concussion. Of the players with multiple concussions, 75 players had two, 23 players had three, 15 players had four, 7 players had 5+. The mean duration between the date of concussion and subsequent concussion was 441 days (SD: 332 days, median: 370 days; range: 11–1435 days) .

The k-means analysis identified six distinct clusters with varying symptom profiles and severities (Table 2). *Cluster 4 Asymptomatic* was the largest (n = 272), characterised by minimal symptom reporting across all domains. *Cluster* 3 All *symptoms* (n = 8) was the smallest cluster and exhibited the highest symptom severity overall, with moderate severity across domains. *Cluster 1 Most symptoms* (n = 14) also displayed broad symptom occurrence, but differed from *Cluster 3* in that symptoms were mostly rated as mild. *Cluster 5 Cognitive/Dizziness* (n = 36) was characterised by mild severity across physical and cognitive symptoms with the highest scores for dizziness, don't feel right, difficulty remembering, feeling slowed down, feeling like in a fog, and difficulty concentrating. *Cluster 6 Sensory/Emotion* (n = 24) was distinguished by elevated blurred vision, pressure in head, headache, sadness, and being more emotional, with comparatively lower cognitive symptom reporting. *Cluster 2 Headache* (n = 76) reported mild-moderate severity, with headache the dominant feature, followed by neck pain, and minimal reporting across other symptoms.

**Table 1:** Included variables from SCRIMMAGE database [13].

Included variables
**Player-level variables**
• Player identifier
• Season (2017–2022)
• Total number of concussions sustained across the study period
**Concussion episode variables**
• Date of concussion diagnosis
• Date of subsequent concussion
• Inter-concussion interval (days between consecutive concussions)
• Number of concussions in the prior two-year window (categorical: 0, 1, 2, 3+)
**SCAT5 symptom variables (22 items, each rated 0**–**6)**
• Headache
• Pressure in head
• Neck pain
• Nausea or vomiting
• Dizziness
• Blurred vision
• Balance problems
• Sensitivity to light
• Sensitivity to noise
• Feeling slowed down
• Feeling in a fog
• Not feeling right
• Difficulty concentrating
• Difficulty remembering
• Fatigue or low energy
• Confusion
• Drowsiness
• More emotional
• Irritability
• Sadness
• Nervous or anxious
• Trouble falling asleep
**Derived symptom variables**
• Total symptom count (number of symptoms endorsed)
• Total symptom severity score (sum of all 22 items, range 0–132)
• Symptom cluster assignment (1–7, derived from k-means)
**Outcome variables**
• Date of medical clearance
• Days to medical clearance
**Temporal variable**
• Assessment date (continuous, centred at mean, used for temporal trend analysis)

**Table 2:**
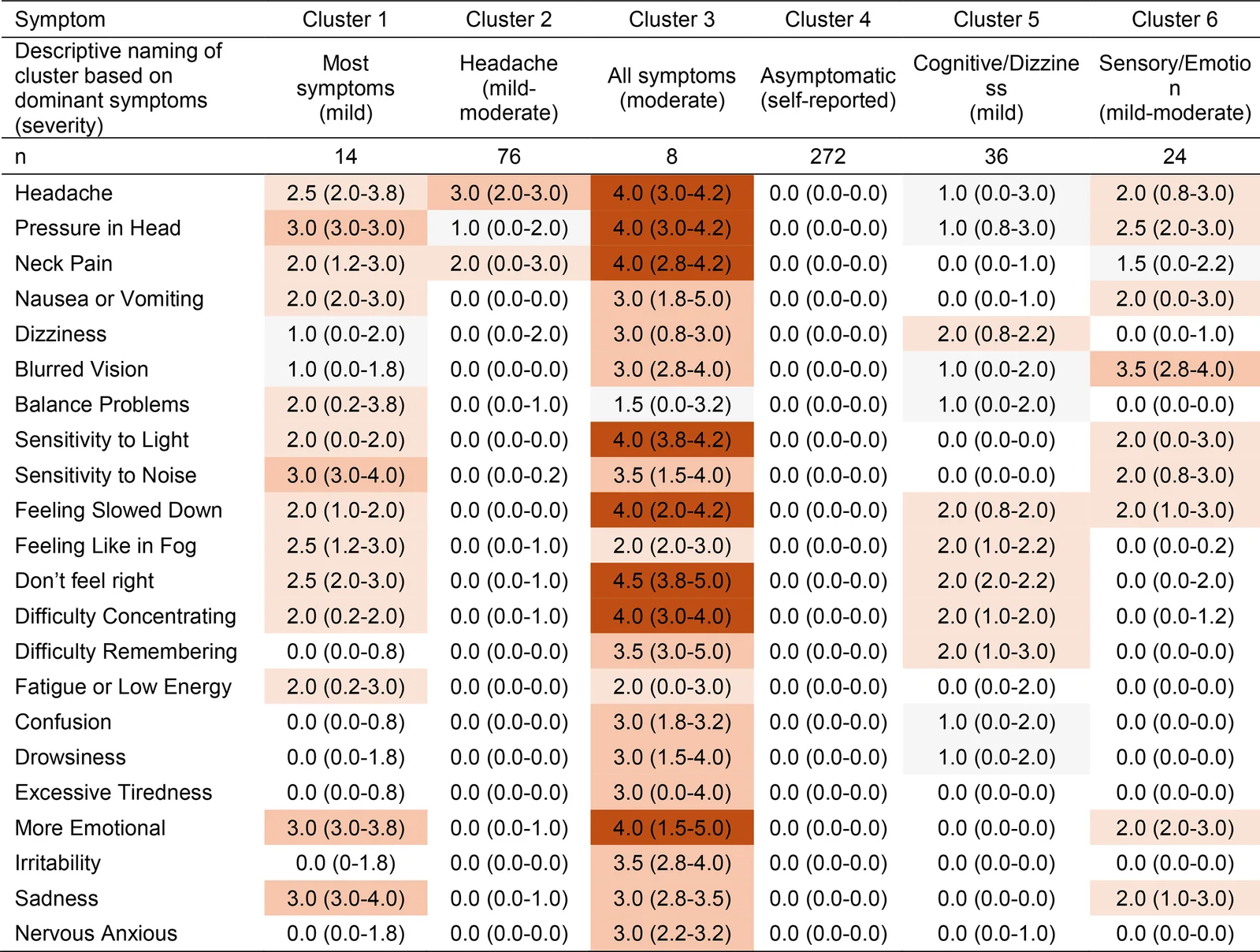
The median and interquartile range of symptom severity for symptom clusters (n = 430 concussions). Possible responses to each symptom ranged from 0 (none) to 6 (severe).

### 3.1 Evaluation of symptom similarity for subsequent concussions

The transition probability matrix revealed distinct patterns of symptom cluster evolution across subsequent concussions. Transition probabilities were calculated from the players who sustained a subsequent concussion; the number of players contributing to each row is given in Table 3. *Cluster 4 Asymptomatic* demonstrated the highest retention of any cluster (p_44_ = 0.80); athletes presenting with few symptoms at initial concussion tended to remain in this cluster following subsequent concussion. *Cluster 1 Most symptoms* and *Cluster* 3 All *symptoms* showed complete transition to *Cluster 4 Asymptomatic* (p_14_ = p_34_ = 1.00), with no retention or transitions to any other cluster observed. *Cluster 2 Headache* also demonstrated a strong transition to *Cluster 4 Asymptomatic* (p_24_ = 0.86), with low retention (p_22_ = 0.07) and a minor transition to *Cluster 1 Most symptoms* (p_21_ = 0.07). *Cluster 5 Cognitive/Dizziness* showed moderate transition to *Cluster 4 Asymptomatic* (p_54_ = 0.65) and a secondary transition to *Cluster 2 Headache* (p_52_ = 0.06); its retention was the highest of any cluster other than Cluster 4 (p_55_ = 0.29). *Cluster 6 Sensory/Emotion* showed no retention with equal probabilities of transitioning to *Cluster 1 Most symptoms or Cluster 2 Headache* (p_61_ = p_62_ = 0.25) and a 50% probability of transitioning to *Cluster 4 Asymptomatic*. Across all clusters, *Cluster 4 Asymptomatic* was the most common destination for a subsequent concussion. Transitions from initial concussion symptom cluster to subsequent concussion symptom clusters are visualised in Fig. 1.

**Table 3:** The probability of a player transferring from one concussion symptom cluster to another following a subsequent diagnosed concussion. Initial concussion symptom cluster is presented in the left hand column (n = 120 players).

		Cluster 1	Cluster 2	Cluster 3	Cluster 4	Cluster 5	Cluster 6
		Most symptoms (mild)	Headache (mild-moderate)	All symptoms (moderate)	Asymptomatic (self-reported)	Cognitive/Dizziness (mild)	Sensory/Emotion (mild-moderate)
Cluster 1	Most symptoms (mild)	0%	50%	0%	50%	0%	0%
Cluster 2	Headache (mild-moderate)	7%	7%	0%	86%	0%	0%
Cluster 3	All symptoms (moderate)	0%	0%	0%	100%	0%	0%
Cluster 4	Asymptomatic (self reported)	4%	9%	1%	80%	6%	0%
Cluster 5	Cognitive/Dizziness (mild)	0%	6%	0%	65%	29%	0%
Cluster 6	Sensory/Emotion (mild-moderate)	25%	25%	0%	50%	0%	0%

**Fig. 1:**
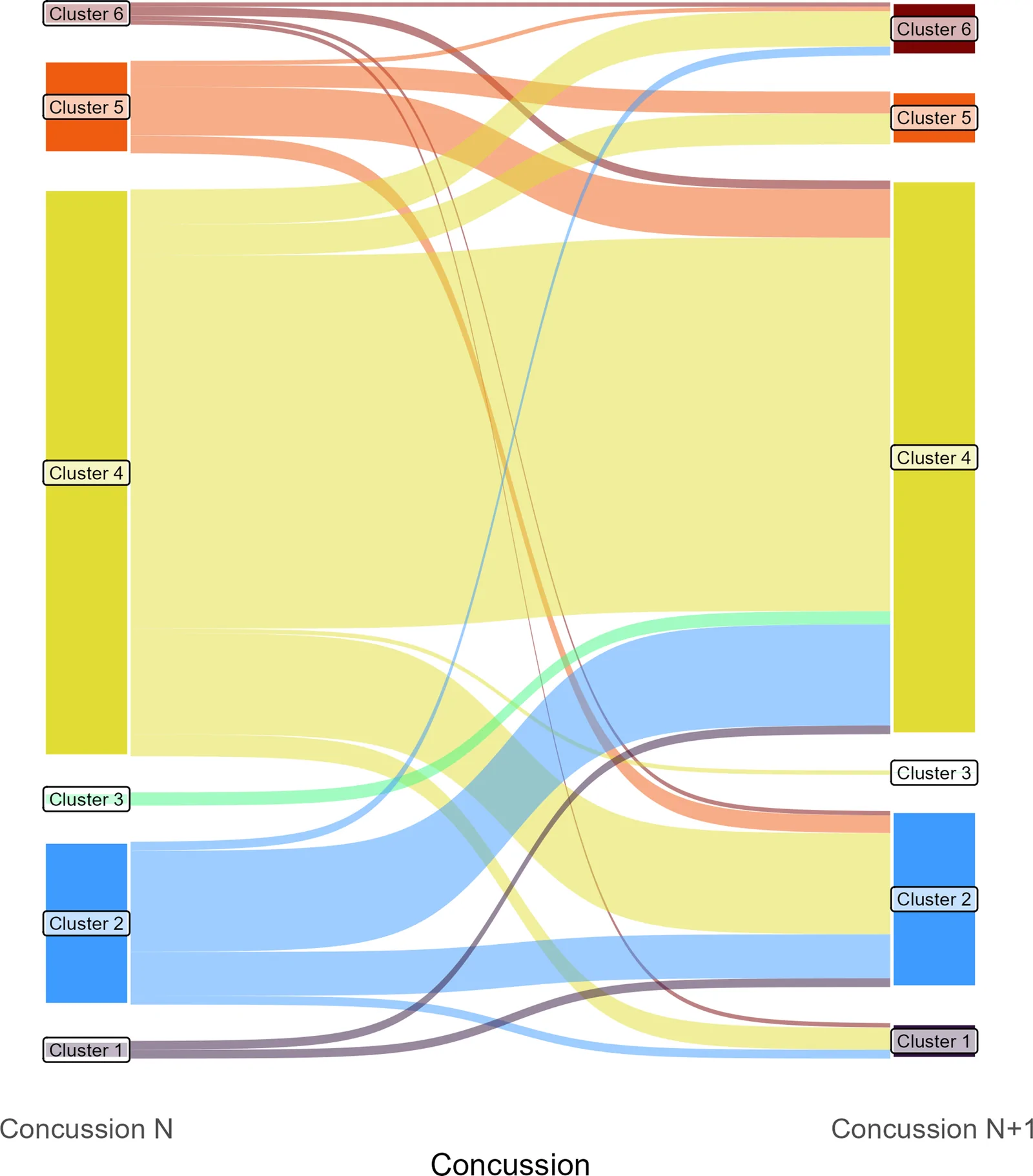
Sankey diagram displaying the transition of players between the 6 symptom clusters.

### 3.2 Relationship between days between concussions and symptom count, and symptom severity

There was no significant association between *days between concussions* and *symptom count* (rate ratio = 1.00, 95% CI [1.00, 1.00], z = −1.62, p = 0.105) or *symptom severity* (rate ratio = 1.00, 95% CI [1.00, 1.00], z = 0.11, p = 0.909) (Fig. 2).

**Fig. 2:**
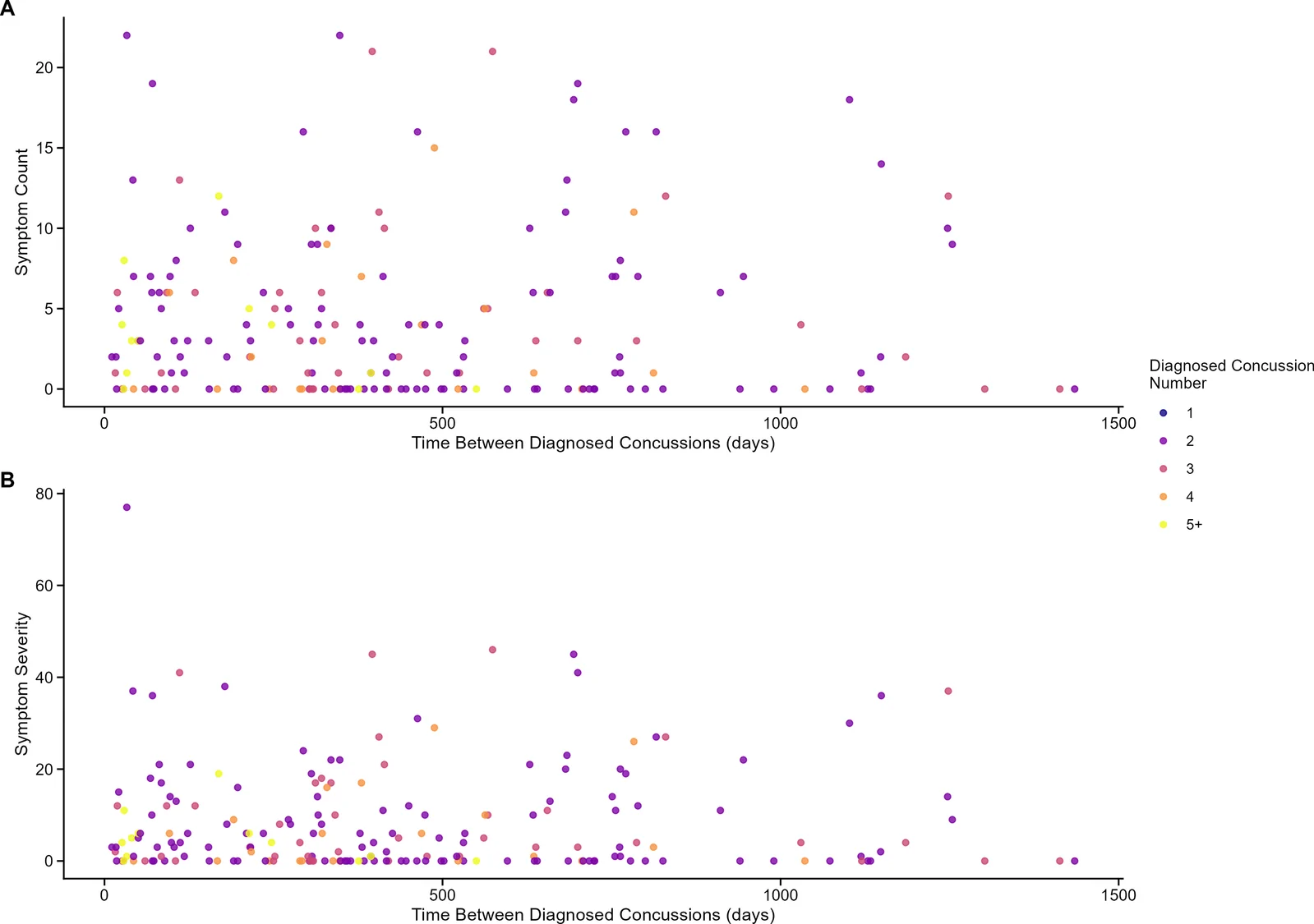
Time between diagnosed concussions plotted against (A) total reported symptom count and (B) total reported symptom severity (Concussions = 319 Players = 120.).

### 3.3 Relationship between days to medical clearance and symptom count, and symptom severity

Figure 3 presents days to medical clearance and the symptom count (A) and severity (B). Symptom count (rate ratio = 1.00, 95% CI [1.00, 1.01], z = 0.99, p = 0.321) was not significantly associated with days to medical clearance, whereas symptom severity was (rate ratio = 1.00, 95% CI [1.00, 1.01], z = 2.37, p = 0.018).

**Fig. 3:**
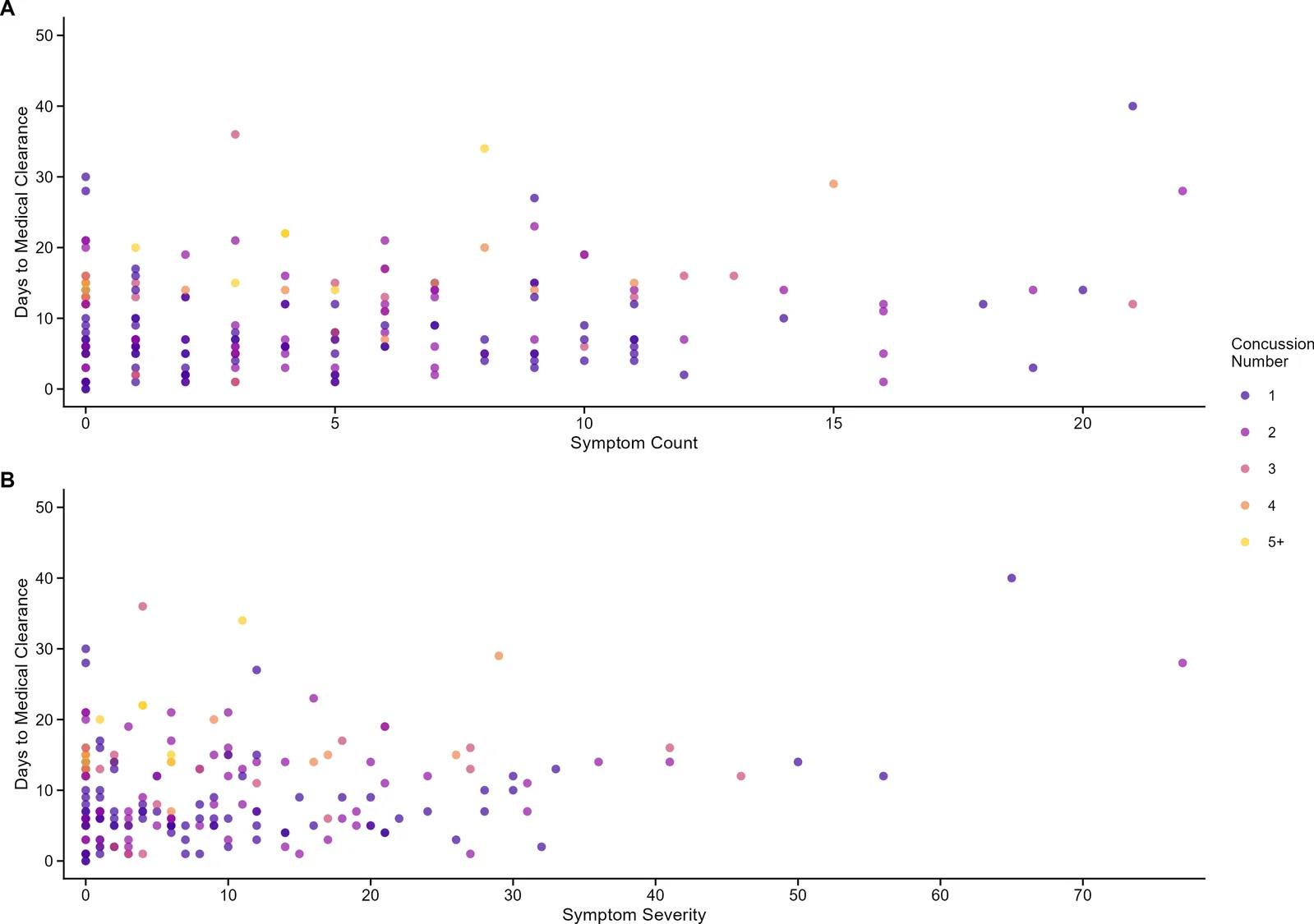
Days to medical clearance plotted against (A) total reported symptom count and (B) total reported symptom severity (Concussions = 212; Players = 106).

### 3.4 Relationship between concussion number and days to medical clearance

Figure 4 presents the median and interquartile range for days to medical clearance according to the number of concussions observed in the prior 2-year window. After adjusting for the change in return to play protocols introduced in 2020, estimated marginal means showed no evidence of an association between clearance time and a higher number of concussions in the preceding two years. Players with no concussions in the prior two years had an estimated marginal mean of 11.6 (95% CI: 9.9, 13.5) days to medical clearance, compared with 12.2 (10.5, 14.2) for one prior concussion, 10.6 (7.9, 14.1) for two prior concussions, and 13.8 (8.3, 23.2) for three or more prior concussions in a two-year period. Pairwise comparisons indicated no significant differences in days to medical clearance between any concussion count grouping (RR range 0.76–1.15, all adjusted p ≥ 0.77).

**Fig. 4:**
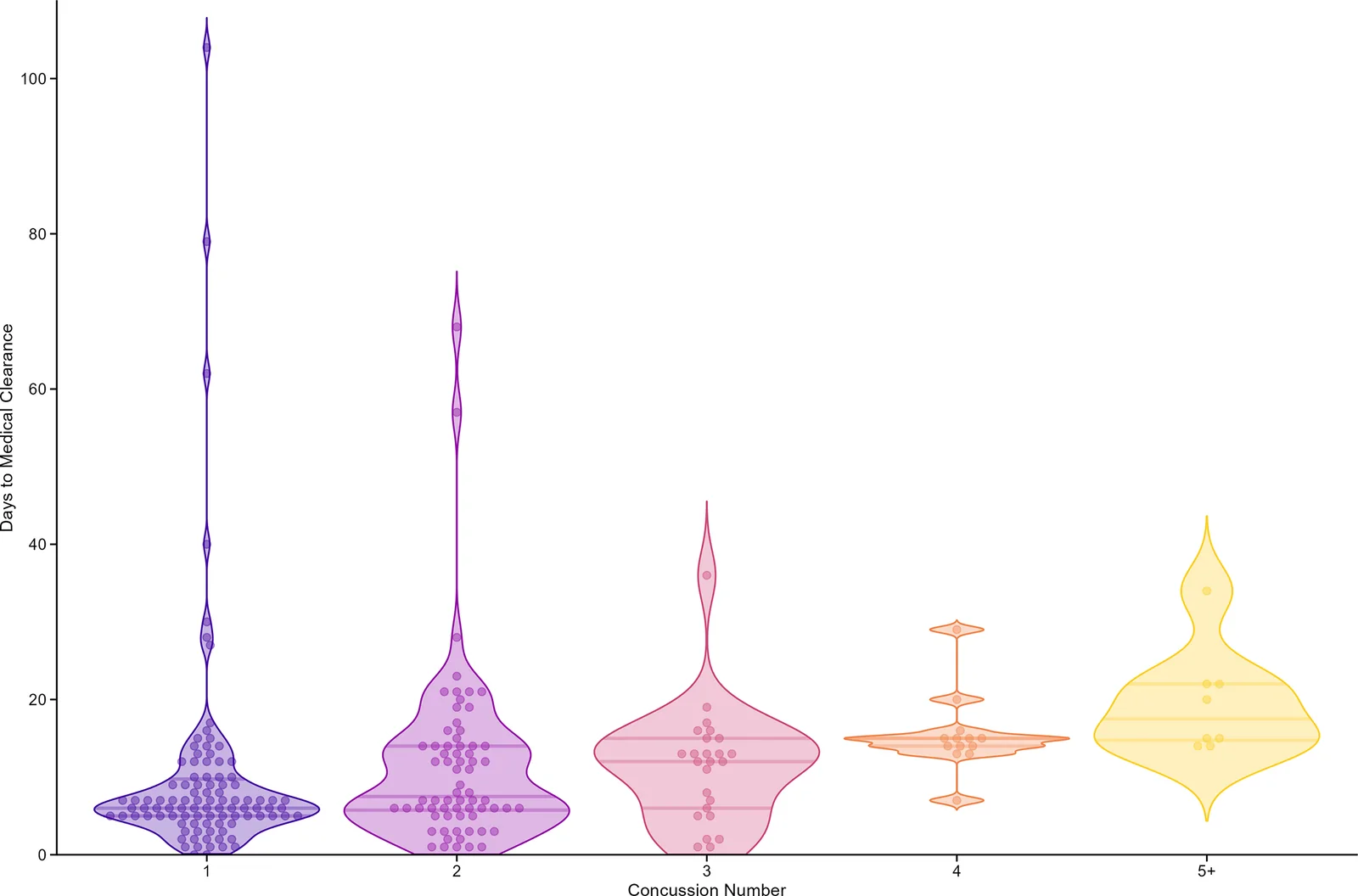
Days to medical clearance for players diagnosed with 0 (n = 102), 1 (n = 106), 2 (n = 25) or 3+ (n = 6) concussions in the prior two-year window. Each circle indicates an individual datapoint, whilst lines on the violin plot indicate the 25th, 50th, and 75th percentile.

### 3.5 Temporal relationship between assessment date and medical clearance

Regression analysis indicated a statistically significant association between concussion assessment date and days to medical clearance (z = 12.8, p < 00.001). At the sample mean assessment date, predicted clearance time was 9.19 days (95% CI: 8.44, 10.00). Assessment date was associated with a small but consistent increase in clearance time across the observation period, with later assessments corresponding to longer recovery durations (Fig. 5). Specifically, each additional calendar year was associated with a rate ratio of 1.36 (95% CI: 1.29, 1.42).

**Fig. 5:**
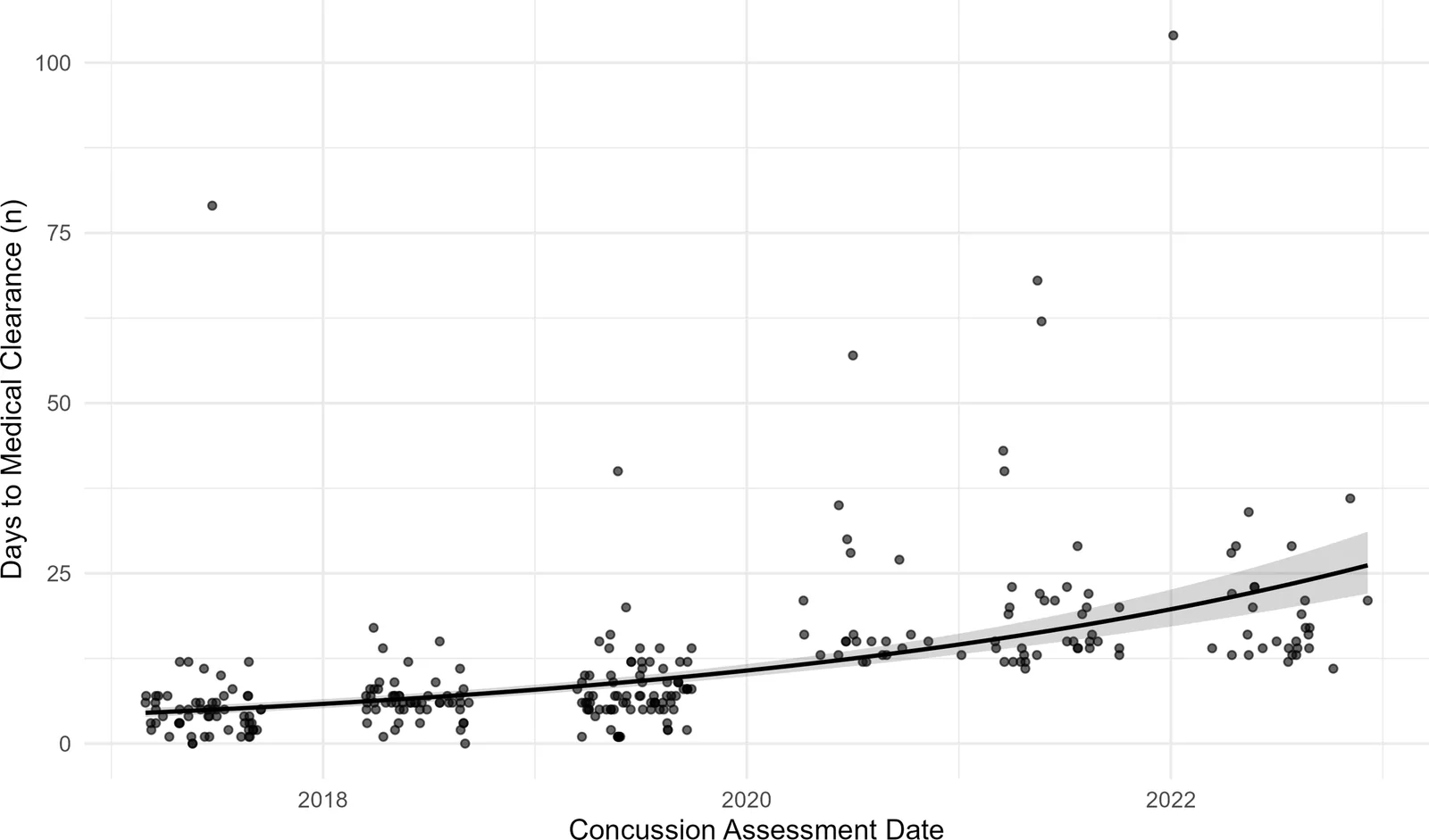
The relationship between date of concussion assessment (2017–2022) and medical clearance (Concussion = 288; Players = 182).

## 4 Discussion

This is one of the largest studies to date reporting on the in-game subjective post-concussion symptom reporting of male professional collision sport athletes with multiple diagnosed concussions. We evaluated the acute symptom reporting of 120 players with multiple concussions over six NRL seasons. Symptom reporting remains an important part of the medical evaluation and understanding the role that acute symptom count and severity may play, could be of clinical significance. We report three main findings. First, players rarely presented in the same symptom cluster twice, with Cluster 4 Asymptomatic the exception: it was both the most retained cluster and the most common destination from all others. Second, symptom count and severity had little relationship to timing: neither was associated with the interval between concussions; only severity was associated with days to medical clearance. Third, days to medical clearance were unrelated to the number of concussions in the preceding two years, but rose by 36% per calendar year across the study period.

Post-concussion symptom research has previously grouped symptoms within four-factor models [8]. Exploratory factor analysis of baseline data from 30,455 high school and collegiate athletes proposed a revised four-factor structure (cognitive-sensory, sleep-arousal, vestibular-somatic, and affective) accounting for 49.1% of the total variance [8]. The same study identified a different four-factor structure following injury (cognitive-fatigue-migraine, affective, somatic, and sleep), indicating that groupings derived at baseline do not necessarily hold post-concussion [8]. Separately, concussion profiles or subtypes have been proposed to characterise the heterogeneity of concussion and to inform targeted clinical management [[16], [17], [18], [19]]. Kontos and colleagues described five clinical profiles (cognitive/fatigue, vestibular, ocular, migraine, and anxiety/mood) with sleep and cervical symptoms treated as modifiers that co-occur across profiles rather than as profiles in their own right [17,18,20]. In a retrospective review of 236 patients presenting to a concussion specialty clinic, migraine (26%) and anxiety/mood (24%) were the most common primary profiles; however, that sample was 58.9% female, spanned ages 11 to 40, and was assessed at a mean of 15.3 days post-injury, limiting direct comparison with acute in-game symptom reporting in male professional athletes [20]. Among the professional male rugby league players in the present study, six acute symptom clusters were identified, with players rarely presenting in the same cluster twice unless that cluster was Cluster 4 Asymptomatic, which was both the most retained cluster and the most common destination from all others. These clusters differ from clinical profiles in derivation, timing and purpose. Clinical profiles are assigned by a clinician on the basis of targeted assessment, including vestibular and ocular motor testing, cervical examination and mood screening, generally undertaken post-acutely in a clinic setting. Our clusters were derived statistically from self-reported symptom items recorded at the time of injury. Clinical profiles are also not mutually exclusive, with athletes commonly assigned primary, secondary and tertiary profiles, whereas our approach assigns each concussion to a single cluster. The symptom content of several clusters resembles the anxiety/mood, cognitive/fatigue, migraine and vestibular profiles, but this correspondence is not sufficient to establish equivalence, and we do not map one set onto the other. Sleep disturbance, which features as a modifier in several profiling frameworks, is not assessed at the acute in-game time point, although drowsiness, fatigue and excessive tiredness are captured. Our findings indicate that acute symptom presentation varies across successive concussions within the same player; sub-acute profiling approaches may therefore need to account for concussion history as a source of variability.

Data were collected across six seasons, a period over which attitudes to head injury and clinical management practices evolved (presented in the settings of methods section). Because such changes could affect how concussions were identified and symptoms reported, we examined whether days to medical clearance varied with concussion assessment date; initial post-concussion symptom severity has been associated with prolonged recovery and longer return to play times [[21], [22], [23], [24]], making clearance time a reasonable index of both injury presentation and management practice. Across the six-season observation period, the time to medical clearance increased by approximately 36% per year. The increase was not uniform: clearance times remained stable at approximately 7 days until 2020, then rose steadily to approximately 21 days by 2022. A similar increase in time to medical clearance in the NRL has been observed in English Rugby Union players over a similar period [25]. Three explanations are plausible but cannot be distinguished with these data. First, return-to-play decisions may have become more conservative independent of the clinical presentation. Second, players may have become less willing to report symptoms. Third, the progressive expansion of video surveillance is likely to have shifted the case mix, such that players displaying video signs that are difficult to detect live were increasingly identified for assessment despite minimal symptom reporting; this would be expected to reduce mean symptom burden across the study period independently of any change in injury characteristics. The third explanation is particularly relevant to the analysis of concussion number, because a player's subsequent concussions necessarily occur later in calendar time than their first, and concussion number is therefore correlated with assessment date. On any of these accounts, concussions in later seasons were recorded under materially different management conditions than those in earlier seasons.

This study has several limitations. First, while the study numbers are large, the number of players with multiple concussions diminished, making the power for some of the analysis weak. Second, the arbitrary decision to truncate the upper limit of exposure to 5 or more concussions may have affected the results of that group by limiting the opportunity to further characterise a dose–response relationship. Third, consideration of the symptom clusters were informed by prior literature and were obtained from a number of studies with small sample sizes. Fourth, two of the six clusters were small (*Cluster 1 Most* symptoms, n = 14; *Cluster* 3 All *symptoms,* n = 8; together 5% of presentations), so their symptom profiles are estimated with limited precision and may not be stable in other samples; the clinical characteristics described for these clusters should therefore be regarded as provisional and require replication in larger cohorts. Fifth, we only used acute post-concussion symptom reporting and did not compare those reports with the baseline symptom reporting, nor serial post-concussion symptom recovery. Sixth, concussion history before the start of the study (2017) was not captured, so a player's first recorded concussion within this dataset may not represent their first career concussion; this incomplete capture of concussion history could confound the classification of injuries as “first” concussions and should be considered when interpreting these findings. Finally, the results may not be generalisable to elite women rugby league players, other levels of match play, other age groups (i.e., children or adolescent rugby league players), or concussed athletes from other sports.

## 5 Conclusion

Subjective symptom reporting has an important role in the medical evaluation of players suspected of having sustained a concussion. In this relatively large sample of professional male rugby league players, who have experienced more than one concussion, the acute subjective symptom reporting varied both between players and within players across successive concussions. We identified six symptom clusters. Subsequent concussions did not typically reproduce the symptom cluster of a previous concussion; rather, players were more likely to transition between clusters, most often towards the cluster characterised by minimal reported symptoms. Symptom severity was associated with the number of days to medical clearance, whereas the number of concussions in the preceding two years was not. Because a substantial proportion of players reported minimal or no symptoms despite a diagnosed concussion and an extended period before medical clearance, the principal clinical implication is that these players require individual clinical assessment, and that management decisions should not rest on acute symptom profiling using the PCSS alone. Increasing time to medical clearance across the study period is likely reflective of evolving management practice rather than as evidence of changing injury severity.

## Ethics declaration

The authors have NOT obtained informed consent from participants or their legal representatives. In accordance with the NRL and Rugby League Players Association (RLPA) Collective Bargaining Agreement, all players consented a priori to the use of deidentified injury data for the use in NRL endorsed research. As such, no individualised written informed consent for the use of a participant's to be included in this study was sought.

This study was performed in compliance with relevant laws, regulatory frameworks and guidelines where the research took place. This study was approved by the University of Newcastle Human Ethics Committee (Approval No. H-2012-0344).

## Declaration of competing interest

MS has no competing interests to declare.

CO has a research fellowship that is part-funded by the Rugby Football League.

LVF is supported through research grant funding at Edith Cowan University. She has previously received project funding from several sports, health and government agencies including: Australian Football League; Australian Institute of Health and Welfare; Australian Institute of Sport; Combat Sport Commission Western Australia; Cricket Australia; Defence Science WA; Exercise & Sports Science Australia; Injury Matters; IOC; KidSafe WA; Rugby Australia; State Government of Victoria; VicSport.

GWF has previously received funding from World Rugby to attend research meetings and was a previous member of the Concussion in Sport expert panel.

BJ has secured research funding from World Rugby, Rugby Football Union, Scottish Rugby, Premiership Rugby, Rugby Football League, Catapult Sports, Prevent Biometrics, HitIQ, Leeds Rhinos Rugby League, Yorkshire Carnegie Rugby Union, Bath Rugby, Wasps Rugby. He is employed in a consultancy capacity by the Rugby Football League, and Premiership Rugby as their research lead.

AJG has a clinical practice in neuropsychology involving individuals who have sustained sport-related concussion. He is a concussion consultant to Rugby Australia and the Sydney Swans Football Club. He is the global clinical lead for the World Rugby Brain Health Service. He is a member of the World Rugby Concussion Working Group, and a member of the Australian Football League Concussion Scientific Advisory Committee. He has received travel funding or been reimbursed by professional sporting bodies, and commercial organisations for discussing or presenting sport-related concussion research at meetings, scientific conferences, workshops, and symposiums. He is supported by a National Health and Medical Research Council (NHMRC) Investigator Grant. He acknowledges unrestricted philanthropic support from the National Rugby League, the International Olympic Committee, the Australian Sports Commission, and World Rugby for research in former elite level athletes.
